# Using Exome Sequencing to Improve Prediction of FOLFIRINOX First Efficacy for Pancreatic Adenocarcinoma

**DOI:** 10.3390/cancers13081851

**Published:** 2021-04-13

**Authors:** Julie Lecuelle, Anne Aarnink, Zoé Tharin, Caroline Truntzer, François Ghiringhelli

**Affiliations:** 1 Platform of Transfer in Biological Oncology, Georges François Leclerc Cancer Center–Unicancer, 1 rue du Professeur Marion, 21000 Dijon, France; jlecuelle@cgfl.fr; 2 UMR INSERM 1231, 7 Boulevard Jeanne d’Arc, 21000 Dijon, France; 3 Department of Medical Oncology, Georges François Leclerc Cancer Center–Unicancer, 1 rue du Professeur Marion, 21000 Dijon, France; aaarnink@cgfl.fr (A.A.); ztharin@cgfl.fr (Z.T.); 4 Genomic and Immunotherapy Medical Institute, Dijon University Hospital, 14 rue Paul Gaffarel, 21000 Dijon, France; 5 University of Burgundy-Franche Comté, Maison de l’université Esplanade Erasme, 21000 Dijon, France

**Keywords:** PDAC, prognostic biomarkers, FOLFIRINOX exome sequencing

## Abstract

**Simple Summary:**

Pancreatic ductal adenocarcinoma is one of the deadliest human cancers. The standard treatment for non-resectable tumors is based on usage of FOLFIRINOX (folinic acid, fluorouracil irinotecan oxaliplatin) chemotherapy. The aim of our retrospective study was to identify genomic markers associated with an improved survival in patients treated with FOLFIRINOX using whole exome sequencing (WES). We successfully generated WES analyses in 78 patients. We created and compared several models with clinical or genomic variables and pathways. While the clinical score was associated with overall and progression-free survival, the genomic score and pathways score were associated with the overall survival. The addition of genomic score improved the prediction of prognosis compared to the clinical score alone. Thus, our study showed that WES could provide useful information to predict survival in patients treated with FOLFIRINOX and might be used to select patients who could yield most benefit from FOLFIRINOX treatment.

**Abstract:**

Purpose: The first line treatment of advanced pancreatic ductal adenocarcinoma cancer (PDAC) comprises a FOLFIRINOX regimen for most patients with good performance status. However, no biomarker to predict efficacy is currently available. We investigated whether exome sequencing could be used to predict progression-free and overall survival in patients undergoing FOLFIRINOX for PDAC. Methods: In this single-center observational study, we included 78 patients with advanced PDAC who underwent somatic and germline exome analyses during first line therapy with FOLFIRINOX or gemcitabine. Exome-derived variables associated with outcome were then used in Cox regression models to generate a composite biomarker. Results: Performance status, tumor stage, liver metastasis, and lung metastasis were retained to generate a prognostic clinical score associated with overall and progression-free survival. Clonality, ploidy, and copy number variant (CNV) signatures 1 and 5, as well as gene variants in the calcium, non-homologous end-joining (NHEJ), and spliceosome pathways, were retained to generate a genomic prognostic score. The addition of genomic score improved the prediction of prognosis compared to the clinical score alone. Conclusions: This study underlines that structural and pathway genomic features could be used to predict FOLFIRINOX survival in patients with advanced PDAC.

## 1. Introduction

Pancreatic ductal adenocarcinoma (PDAC) is one of the deadliest human cancers. Incidence in 2021 is expected to be around 43,000 cases in Europe, with predicted rates of 8.1/100,000 in men and 5.6/100,000 in women [1]. Five-year overall survival has modestly improved in the past few decades, which in contrast with many other human solid malignancies.

The best treatment is surgical resection, which is possible only at an early stage, and offers a median survival of 55 months with FOLFIRINOX chemotherapy and 35 months with gemcitabine [2]. The poor prognosis of PDAC is notably explained by diagnosis at a late stage in most patients [3], as well as by the lack of effective treatments to control the disease [4]. 

Since 2011, the standard treatment for non-resectable PDAC has been a combined chemotherapy regimen including oxaliplatin, irinotecan, fluorouracil, and leucovorin (FOLFIRINOX), which has shown an improvement in median overall survival compared to gemcitabine (11.1 months versus 6.8 months, respectively). However, about 30% of patients experience rapid progression under FOLFIRINOX, and this regimen can be highly cytotoxic [5]. In 2013, a phase III trial compared the efficacy and safety of the addition of nab-paclitaxel to gemcitabine versus gemcitabine monotherapy [6]. Overall survival was significantly improved (8.5 versus 6.7 months, respectively), as were progression-free survival and response rate, but with increased neuropathic and hematologic toxicity. The combination of nab-paclitaxel plus gemcitabine has become an option for first-line therapy in North America [6]. 

Other research efforts in recent years focused on developing new treatments, such as targeted therapies and immunotherapies, have unfortunately yielded disappointing results. Recent studies have revealed different subtypes of early-stage PDAC that could have potential therapeutic implications [7,8,9]. The genomic characterization of advanced PDAC may identify clinically relevant alterations that could be leveraged to improve the management of metastatic pancreatic disease [10]. However, only a small subset of patients with PDAC presenting certain aberrations can benefit from novel therapies [11], most notably germline BRCA (breast cancer) 1/2 mutations [12], mismatch repair (MMR) deficiencies [13], and NTRK1-3 (neurotrophic tyrosine kinase receptor) fusions [14]. Therefore, chemotherapy remains the mainstay of treatment for patients with advanced PDAC. Currently, no biomarkers are available to identify patients who are most likely to benefit from an aggressive chemotherapy approach and those who will likely yield little benefit due to toxicities. Using whole exome sequencing (WES) data, the aim of our single-center, retrospective cohort study was to identify genomic markers associated with improved survival in patients treated with FOLFIRINOX for advanced PDAC. 

## 2. Materials and Methods

### 2.1. Study Population 

We included 78 patients with locally advanced or metastatic PDAC in whom WES analysis was performed as part of routine care and then interpreted by the molecular tumor board of the Georges François Leclerc Cancer Center. The inclusion criteria for exome analysis were validated by the tumor board for each patient. All patients who needed to be included in first or second line of treatment and present a performance status of 0 or 1. Patients were followed between January 2010 and September 2020. WES analyses were performed on the initial diagnostic biopsy. The assessment of best response was based on radiological imaging in line with the response evaluation criteria in solid tumors (RECIST) 1.1 criteria.

### 2.2. Sample Selection

The WES analysis was performed in routine care in our center in order to identify potentially targetable mutations for second-line therapy. Before patients consented to WES of their tumoral tissue, they were informed by their oncologist. Physicians selected an archival tumor sample (primary or metastasis) for genomic analysis. At the physician’s discretion, a new tumor biopsy could be proposed to the patient. Germline testing was performed after counseling by a clinical geneticist. 

Only patients for whom written informed consent was obtained and recorded in the medical chart were included in this study. The study was approved by the CNIL (French national commission for data privacy) and the local ethics committee, and it was performed in accordance with the Helsinki Declaration and European legislation.

Only 11 patients received treatment based on molecular tumor board recommendation. Only one patient yielded a significant clinical benefit from this strategy with more than 6 months progression-free survival (PFS) (Supplementary Table S1). This patient presented biallelic deficiency in the BRCA2 gene and was treated with PARP (poly [ADP-ribose] polymerase) with immunotherapy in clinical trial.

### 2.3. DNA Isolation

DNA was isolated from archival tumor tissue using the Maxwell 16 FFPE Plus LEV DNA purification kit (Promega, Madison, WI, USA). DNA from whole blood (germline DNA) was isolated using the Maxwell 16 Blood DNA Purification kit (Promega), following the manufacturer’s instructions. The quantity of extracted genomic DNA was assessed by a fluorometric method with a Qubit device.

### 2.4. Whole Exome Capture and Sequencing

Two hundred nanogram of genomic DNA were used for library preparation by using the Agilent SureSelectXT reagent kit (Agilent Technologies, Santa Clara, CA, USA). The totality of the enriched library was used in the hybridization and captured with the SureSelect All Exon v5 or v6 (Agilent Technologies) baits. Following hybridization, the captured libraries were purified according to the manufacturer’s recommendations and amplified by polymerase chain reaction (12 cycles). Normalized libraries were pooled, and DNA was sequenced on an Illumina NextSeq500 device using 2 × 111-bp paired-end reads and multiplexed. More than 90% of the target sequence were covered with a read depth of at least 10X for somatic DNA.

### 2.5. Exome Analysis Pipeline

Reads in the FASTQ format were aligned to the reference human genome GRCh37 using the Burrows–Wheeler aligner (BWA v.0.7.15). Local realignment was performed using the Genome Analysis Toolkit (GATK v.3.6). Duplicate reads were removed using Picard v.2.5. To identify somatic single-nucleotide variants (SNVs), a validated pipeline that integrated mutation calls from three different mutation callers was used. SNVs were called with VarScan (v2.4.3) [15] and Mutect (v1.1.7) [16], and insertion/deletions (indels) were called with VarScan and Strelka (v2.9.2) [17]. Tumor mutational burden (TMB) was calculated using the number of significant SNVs (with Untranslated Transcribed Region, synonyms, introns, and intergenic SNVs filtered out) divided by the number of megabases covered at a defined level. To identify tumor-specific mutant peptides, pVAC-Seq v4.0.3 [18] (personalized *V*ariant *A*ntigens by *C*ancer *Seq*uencing) was used; pVAC-Seq is based on human leukocyte antigen (HLA) typing obtained by HLAminer [19]. TITAN [20] was used to infer the number of copy number alteration (CNA) subclones, the number of large deletions, and the loss of heterogeneity (LOH) > 15 Mb from whole-exome sequencing data. It was also used to estimate tumor ploidy. SNV signatures were generated using DeconstructSigs (v1.8.0) [21] and COSMIC signatures identified by Alexandrov et al. [22].The weighted combination of these 30 published signatures was estimated so that, when summed, it most closely reconstructed the somatic mutational profile of each patient. Copy number variant (CNV) signatures were inferred following the methodology of Macinthyre et al. [23]. The copy number profile of each patient was reconstructed based on the weighted combination of 7 signatures. The microsatellite instability (MSI) score was computed using MSIsensor [24]. The homologous recombination deficiency (HRD) score was obtained through the scarHRD [25] pipeline.

### 2.6. Statistical Analysis

Patient and disease characteristics were compared by treatment group using the chi-squared or Fisher’s exact test for qualitative variables, as well as the Wilcoxon test for continuous variables, as appropriate.

Continuous variables were dichotomized using the methodology of Lausen et al. via the maxstat library [26]. For each variable, the first group content at least 30% of the population was used. For survival analysis, the prognostic value of the different variables was tested using univariate or adjusted multivariate Cox regression models with lasso penalty for PFS or overall survival (OS). Survivors were removed from the study at 24 months. Survival probabilities were estimated using the Kaplan–Meier method, and survival curves were compared using the log-rank test. Different survival models were estimated: one with clinical variables only, one with genomic variables only, another with pathways, two combined models with clinical and genomic variables (or pathways), and a last one combining all types of variables (Supplementary Figure S1).

For each of these models, variables were selected for inclusion in the Cox regression models with lasso penalty. To limit optimism and overfitting, 100 bootstrap samples were generated, and only variables mostly (>90%) selected through the 100 corresponding lasso Cox models were kept in the final models. This procedure allowed us to limit optimism and overfitting. Supplementary Table S2 presents corresponding mean hazard ratios with confidence intervals based with 5th and 95th percentiles.

In a second step, composite variables were estimated based on the linear predictor of the corresponding Cox models. Dichotomized scores were estimated based on these composite variables (low vs. high) using the best cutoff strategy based on the library maxstat [26]. A prognostic biomarker is defined as a measurement that is associated with clinical outcome regardless of the treatment, while a predictive biomarker is defined as a measurement that is associated with response to a specific treatment.

Nested multivariate models were compared using the likelihood ratio test (LRT).

Statistical analyses were performed using the R software (http://www.R-project.org/) (version 4.0.2, accessed on 22 June 2020) and graphs were drawn using GraphPad Prism version 7.03.

## 3. Results

### 3.1. Patients’ Clinical Characteristics

Sixty-six patients were treated in first line therapy by FOLFIRINOX, and 12 were treated by gemcitabine. Comparing clinical characteristics between gemcitabine and FOLFIRINOX groups revealed that there were no significant differences, with the exception of response rate, which was better in the FOLFIRINOX group (Table 1). 

### 3.2. Patients’ Genomic Characteristics:

**Figure 1:**
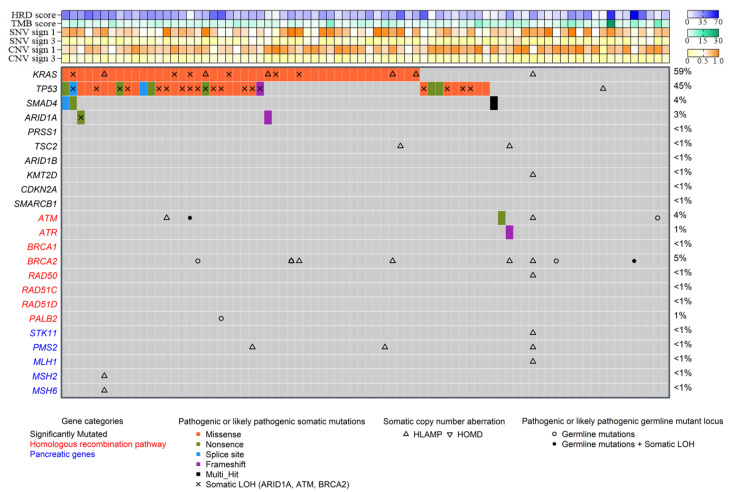
Landscape of genomic mutations in the whole population (*n* = 78 samples). Only pathogenic or likely pathogenic somatic and germline mutations are presented in this landscape. For somatic mutations, we selected those most frequent in the whole cohort, genes involved in the homologous recombination pathway, and genes known to be characteristic of pancreatic tumor. Information about somatic copy number alterations was added. The LOH is shown for all germline mutations but only for some somatic mutations (ARID1A, ATM, and BRCA2). The percentages of patients carrying a somatic or germline mutation in the cohort are shown at the left. At the top, for each sample, expressions of SNV and CNV signatures 1 and 3 are shown, as well as the TMB without splicing regions and HRD scores. TMB: tumor mutational burden; HRD: homologous recombination deficiency; SNV: single-nucleotide variants; CNV: copy number variant; HLAMP: high level amplification; HOMD: homozygous deletion; LOH: loss of heterozygosity.

Patients’ genomic characteristics are described in Table 1. The two most frequently represented SNV and CNV signatures were signatures 1 and 3 (Figure 1 and Supplementary Figure S2A,B). From a correlation matrix, we observed that TMB, neoantigen, and MSI score were strongly correlated. Supplementary Figure S2C shows the correlation between all genomic characteristics. The five most frequently mutated genes were TP53, KRAS, KMT2D, PRSS1, and CDKN2A (Figure 1 and Supplementary Figure S2D). When pooling mutations in pathways, the most substantially mutated pathways were related to the Extracellular signal-regulated kinases (ERK), chromatin organisation, p53, and Receptor tyrosine kinases (RTK) pathways (Supplementary Figure S2E and the description of genes in interest is in Supplementary Table S3). The FOLFIRINOX and gemcitabine cohorts did not differ in terms of WES variables. No significant difference was observed for OS and PFS between patients with KRAS wild-type tumors and patients with KRAS-mutated tumors (respectively hazard ratio (HR) = 1.2 [0.67;2.15], *p* = 0.54; and HR = 0.73 [0.39;1.36], *p* = 0.32). 

The HRD score, SNV3, and CNV3 are known to be associated with the presence of mutations or deletions of homologous repair. In this cohort, these parameters were not found to be correlated (Supplementary Figure S2C). To further investigate, we tested the level of these three variables in patients with or without genetic aberrations in genes that had some activity within the HR-DDR (homologous recombination DNA damage repair) pathway: ARID1A, ATM, ATRX, BAP1, BARD1, BLM, BRCA1/2, BRIP1, CHEK1/2, FANCA/C/D2/E/F/G/L, MRE11A, NBN, PALB2, RAD50, RAD51C, and RAD51B [27,28]. We defined HRD-altered samples based on the presence of biallelic damages with either germline or somatic mutations (null, truncating, and splice-site variants), as well as presence of one mutation on one allele and/or the deletion on the other allele (Supplementary Figure S3). The CNV3 and HRD scores were significantly higher in patients with monoallelic HRD gene variation compared to wild-type (WT) patients. For patients with the biallelic HRD gene variation SNV3, CNV3 and HRD seemed numerically higher than for WT patients, but differences were not significant because of the rarity of the events. Values of each parameter overlapped between the three groups, thus illustrating the difficulty of determining the optimal cut-off values defining these groups. However, we observed that for biallelic mutations, the SNV3 and CNV3 scores were more discriminating. Moreover, in our cohort, four patients had germline mutations in the BRCA2 genes, and mutation status was not correlated with the HRD score (Wilcoxon test: *p* = 0.53).

### 3.3. Association of Clinical and Genomic Variables with Response to FOLFIRINOX

For the FOLFIRINOX group, on the basis of Cox univariate analysis, poor performance status, the presence of liver metastasis, and stage IV diseases were all associated with a poor OS. The presence of lung metastasis was associated with improved survival; similar results were observed for PFS (Table 2). For structural genetic variables, only polyploidy was associated with poor outcome, while polyclonality was associated with good survival. No SNV signature was associated with survival. Only a high expression of CNV signature 1 (>86.6%) was associated with a better overall survival, while an expression of CNV signature 5 greater than 4.7% was associated with a poor survival (Table 2).

**Figure 2:**
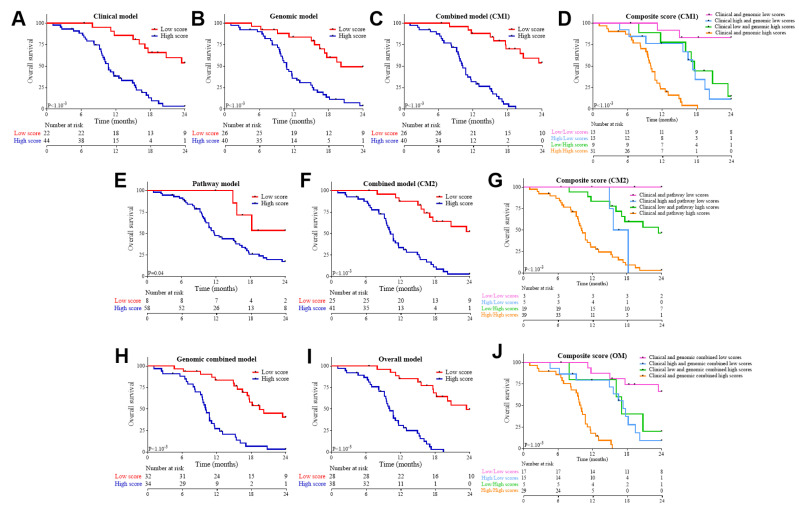
Description of the clinical, genomic, pathway, and combined models for overall survival in the FOLFIRINOX group. Kaplan–Meier curves for overall survival with patients stratified according to the clinical score (**A**), the genomic score (**B**), the combined model 1 (CM1) score (**C**), the low vs. high score (a composite score combining clinical and genomic scores) (**D**), the pathway score (**E**), the combined model 2 (CM2) score (**F**), a composite score combining clinical and pathway scores (**G**), the genomic combined score (genomic variables and pathways) (**H**), the overall score (**I**), and a composite score combining clinical and genomic combined scores (**J**).

**Figure 3:**
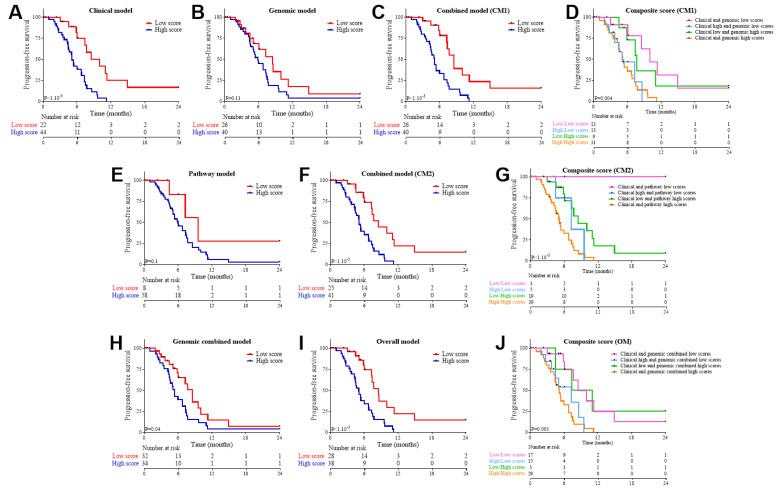
Validation of the overall survival models on progression-free survival in the FOLFIRINOX group. Kaplan–Meier curves for progression-free survival with patients stratified according to scores estimated through overall survival models: the clinical score (**A**), the genomic score (**B**), the CM1 score (**C**), the low vs. high score (a composite score combining clinical and genomic scores) (**D**), the pathway score (**E**), the CM2 score (**F**), a composite score combining clinical and pathway scores (**G**), the genomic combined score (genomic variables and pathways) (**H**), the overall score (**I**), and a composite score combining clinical and genomic combined scores (**J**).

Multivariate models were generated using Cox regression with lasso penalty. The following variables were selected: (1) performance status, tumor stage, and liver and lung metastasis for the clinical model; (2) clonality, ploidy, and CNV signatures 1 and 5 for the genomic model. The model combining both clinical and genomic variables was here called combined model 1 (CM1). Based on dichotomized clinical and structural genomic scores, we separated patients into two groups according to their respective scores. The clinical model was valuable to predict both OS and PFS, while the genomic model was only valuable to predict OS (Figure 2A,B for OS and Figure 3A,B for PFS). The CM1 model predicted both OS and PFS (Figure 2C or Figure 3C).

The CM1 (area under the curve (AUC)= 0.94) had better predictive ability than the clinical (AUC = 0.9; LRT: *p* = 0.05) or genomic (AUC = 0.83; LRT *p* < 1.10^−3^) models alone. To further investigate, we estimated a composite variable by combining clinical and genomic scores. In the case of OS (Figure 2D), the clinical and genomic scores both added complementary information and made it possible to stratify patients into four groups, and the use of both types of variables was required to refine patient prognosis. In the case of PFS, genomic information did not add any incremental predictive value to the clinical score (Figure 3D). 

To further investigate, we pooled mutated genes into KEGG pathways. A pathway was considered mutated if one gene involved in this pathway was mutated. We observed that mutated calcium pathways were associated with a good overall survival, while mutations in Class I MHC (major histocompatibility complex), MAPK (mitogen-activated protein kinase), NHEJ, p53, spliceosome, VEGF (vascular endothelial growth factor) pathways were associated with a poor outcome. The calcium, class I MHC, MAPK, spliceosome, and VEGF pathways had similar impacts on PFS (Table 2). Cox regression models with lasso penalty selected calcium, NHEJ, and spliceosome to generate a prognostic pathway model. The pathway model and the model combining clinical and pathway scores (CM2) were able to predict OS (Figure 2E,F). Moreover, by using time-dependent AUC, we observed that the combined model (CM2) significantly improved the clinical-alone model (AUC = 0.91; LRT: *p* = 0.001) and the pathways-alone model (AUC = 0.65; LRT: *p* < 1.10^−3^). This was further illustrated by estimating a composite score based on the combination of clinical and pathway scores (Figure 2G). This final model was able to predict PFS, contrary to the pathway model (Figure 3E,F–G). 

Finally, an “overall” model (OM) was constructed by combining CM1 and CM2. We were thus able to separate patients into two groups according to a score combining clinical, pathway, and structural genomic information (Figure 2H). The overall model was highly predictive of OS (Figure 2I), and both parts of the overall model (clinical as well as combined genomic scores) were useful to refine OS prediction (Figure 2J). The LRT underlined that CM2 was significantly better (*p* = 0.15). The OM was also valuable to predict PFS (Figure 3H–J).

### 3.4. Association of Clinical and Genomic Variables with Response to Gemcitabine 

To further determine the prognostic versus predictive value of clinical and genomic scores, we tested these scores on the gemcitabine cohort. While the clinical model was highly prognostic of OS in this cohort, neither the structural genomic score nor the pathway score had prognostic value (Supplementary Figure S4). Considering the low number of patients, this observation might suggest that clinical variables are mainly prognostic and genomic variables mainly predictive. 

## 4. Discussion

This study unraveled the capacity of WES sequencing to predict survival in patients treated in first-line by FOLFIRINOX for locally advanced PDAC. We underlined that classical clinical prognostic variables are prognostic for patients treated with either FOLFIRINOX or gemcitabine. However, some variables extracted from the WES seemed to yield added prognostic value and might be more predictive than prognostic. 

An important feature of our analysis was that the gene variants considered in pathways are valuable predictive markers associated with outcome in metastatic disease. Most reports performed in localized pancreatic cancer to define prognostic groups have generated groups based on transcriptomic features mutation features [7,8,9]. For metastatic disease, the Study of Changes and Characteristics of Genes in Patients With Pancreatic Cancer for Better Treatment Selection (COMPASS) showed that chemotherapy responses differed among patients with different tumor subtypes [29]. Collisson et al. led a study that described three intrinsic subtypes of PDAC that clinically progress at different rates and may respond differently to selected therapies [9]. Here, we showed that when involved in pathways, mutations could also provide valuable incremental information.

Most genomic studies have focused on proposing target therapies [10]. Humphris et al. analyzed a total of 385 sporadic PDAC patient samples with large genomic analysis [30]. Only 20 patients (5%) were characterized by a high mutation load (≥4 somatic mutations per megabase), and five were considered hypermutated (≥12 somatic mutations per megabase). Four mismatch-repair (MMR)-deficient tumors (1% of the total) were detected, and all were hypermutated. For the remaining 15 patients with a high mutation burden, the major cause of mutational load was homologous recombination repair (HRR) deficiency. However, these data were not prognostic. A study by Connor et al. attempted to translate genomic sequencing analyses into clinical benefits [31]. The whole-genome sequencing of 160 samples was performed, and four major PDAC subtypes were identified using SNV signatures. Despite this classification, the molecular subtyping was of no prognostic significance and was not associated with tumor pathologic features [31]. Importantly, homologous repair deficiency was not associated with improved response to platinum-based chemotherapy [31].

In our cohort, we also observed that the HRD score was not associated with prognosis. Unexpectedly, in our study, we did not observe any correlations between CNV3, SNV3 signature, and HRD score. This discrepancy may have been due to the fact that metastatic PDAC tumors behave differently or due to technical issues related to depth of sequencing or use of paraffin embedded sections. However, when looking at HRD mutations, we could link mutations with a high level of HDR or CNV3 signature, thus suggesting that these variables are probably the best surrogate markers of homologous repair deficiency in advanced PDAC. Recent data showed that patients with tumors harboring mutations in genes involved in DNA damage repair are likely to have greater benefit from FOLFIRINOX [32]; in contrast, we could not confirm this observation in our study.

Surprisingly, we observed that SNV signatures did not have prognostic value. In contrast, CNV signatures, especially high CNV1 and polyclonality, were associated with a good prognosis. For the CNV1 signature, these findings corroborated those reported in ovarian cancer, where the CNV1 signature (related to mutations in the MAPK pathway) was also related to better outcome [23]. For polyclonality, our findings contradicted many reports in various cancers that have linked polyclonality with resistance to chemotherapy [33]. 

In contrast, polyploidy was related to a poor prognosis; a possible explanation may be the strong correlation between polyploidy and the CNV5 signature, related to chromothriptic-like events. Previous reports have underlined that chomothripsis and polyploidy could account for PDAC aggressiveness [34,35]. 

Cancer genomics traditionally focuses on the characterization of individual molecular mechanisms that can contribute to cancer, such as single nucleotide variations. Due to the multiple levels of genomic and non-genomic heterogeneity, such information is classically poorly predictive for clinics. In recent years, research has underlined that cancer evolution involves a genome reorganization-mediated punctuated phase and chromosomal evolution. Such genome chaos has been commonly detected in various cancers and is linked to the metastasis process and drug resistance [36]. For example, in lung cancer, intra-tumor heterogeneity mediated through chromosome instability was found to be associated with an increased risk of recurrence or death [37]. Our results on the prognostic role of polyploidy thus link with previous reports and enforce the importance of studying chromosomal features—not only punctual variations.

The main limitations of our study were the low number of patients and its retrospective nature. The modest number of patients in the FOLFIRINOX cohort led to the softening of our conclusion and the consideration of the results from an explorative point of view that needs further confirmation in the future through prospective validation. The weak number of patients in the gemcitabine group and the clinical difference between patients who received first line FOLFIRINOX or gemcitabine may have induced some bias. In addition, WES analysis was performed on paraffin-embedded sections taken at the time of diagnosis, before the initiation of treatment, and in different hospitals. All these points may have impaired the homogeneity and quality of data. Accordingly, despite their interest, our results warrant prospective validation in external cohorts.

## 5. Conclusions

In conclusion, our study showed that WES could provide useful information to predict survival in patients treated with FOLFIRINOX. Important information can be gleaned from structural information and global pathway analysis, suggesting that whole genome sequencing or WES could be used to select patients who could yield the most benefit from FOLFIRINOX treatment.

## Figures and Tables

**Table 1:** Summary of clinical and genomic characteristics of 78 patients with locally advanced or metastatic pancreatic ductal adenocarcinoma cancer (PDAC) who underwent whole exome sequencing (WES).

Variables		Whole Population (*n* = 78)	FOLFIRINOX Group (*n* = 66)	Gemcitabine Group (*n* = 12)	*p*-Value
**Clinical characteristics**	Sex, n (%)				0.53
	*Men*	41 (52.6%)	36 (54.5%)	5 (41.7%)	
	*Women*	37 (47.4%)	30 (45.5%)	7 (58.3%)	
	Who performance status, n (%)				0.35
	*0*	30 (38.5%)	27 (40.9%)	3 (25%)	
	*1 and 2*	48 (61.5%)	39 (59.1%)	9 (75%)	
	Stage, n (%)				0.51
	*III*	25 (32.1%)	20 (30.3%)	5 (41.7%)	
	*IV*	53 (67.9%)	46 (69.7%)	7 (58.3%)	
	Lung metastasis, n (%)				0.28
	*No*	60 (76.9%)	49 (74.2%)	11 (91.7%)	
	*Yes*	18 (23.1%)	17 (25.8%)	1 (8.3%)	
	Liver metastasis, n (%)				0.13
	*No*	36 (46.2%)	33 (50%)	3 (25%)	
	*Yes*	42 (53.8%)	33 (50%)	9 (75%)	
	Peritoneal metastasis, n (%)				0.72
	*No*	57 (73.1%)	49 (74.2%)	8 (66.7%)	
	*Yes*	21 (26.9%)	17 (25.8%)	4 (33.3%)	
	Lymph node metastasis, n (%)				0.06
	*No*	59 (77.6%)	47 (73.4%)	12 (100%)	
	*Yes*	19 (22.4%)	17 (26.6%)	0	
	*Missing data*	2	2	0	
	Age at diagnosis, median (IQR)	67.41 (13.82)	67.41 (11.7)	68.41 (22.78)	0.83
	Age at diagnosis, n (%)				0.5
	*≤60 years*	24 (30.8%)	19 (28.2%)	5 (41.7%)	
	*>60 years*	54 (69.2%)	47 (71.2%)	7 (58.3%)	
	Response rate, n (%)				0.02
	*Partial or complete response*	22 (28.2%)	22 (33.3%)	0	
	*Stabilization or progression*	48 (61.5%)	37 (56.1%)	11 (91.7%)	
	*Missing data*	8 (10.3%)	7 (10.6%)	1 (8.3%)	
	OS, median (IQR)	14.93 (11.14)	14.93 (11.14)	14.13 (14.9)	
	PFS, median (IQR)	5.97 (4.4)	6.77 (4.14)	4.77 (9.17)	
**Genomic CharacteristCs**	MSI categories, n (%)				
	*MSS (score ≤ 5)*	77 (98.7%)	65 (98.5%)	12 (100%)	1
	*MSS low and high*	1 (1.3%)	1 (1.5%)	0	
	TMB without splicing regions categories, n (%)				
	*Low (score ≤ 40)*	75 (96.2%)	63 (95.5%)	12 (100%)	1
	*High*	3 (3.8%)	3 (4.5%)	0	
	MSI score, median (IQR)	0.04 (0.14)	0.04 (0.13)	0.02 (0.25)	1
	TMB without splicing regions score, median (IQR)	3.3 (2.01)	3.2 (1.98)	3.8 (2.02)	0.11
	HRD score, median (IQR)	21 (17)	21 (18.5)	21 (13.75)	0.71
	Ploidy, median (IQR)	2 (0.2)	2 (0.27)	2 (0.22)	0.43
	Clonality, median (IQR)	1 (1)	1 (1)	1 (1)	0.74
	TCR clones, median (IQR)	5 (4)	5 (3)	7.5 (8.25)	0.23
	Deletions with LOH, median (IQR)	4 (14.75)	4 (14.75)	5.5 (13.5)	0.72
	Microdeletions, median (IQR)	3 (6)	3 (6)	2.5 (5.25)	0.87
	Neoantigens, median (IQR)	5 (5)	5 (5)	5 (3)	0.48
	Strong neoantigens, median (IQR)	1 (1)	1 (1)	0.5 (1.25)	0.96

Continuous variables were described by median values and interquartile range (IQR) and compared between cohorts with Wilcoxon test. Categorical variables were described by number of observation (%) and compared between cohorts with Fisher’s exact test for count data. MSI: microsatellite instability; MSS: microsatellite stable; TMB: tumor mutational burden; HRD: homologous recombination deficiency; TCR: T cell receptor; OS: overall survival; PFS: progression-free survival; LOH: loss of heterozygosity.

**Table 2:** Univariate Cox model for overall and progression-free survival in the FOLFIRINOX cohort. For each type of variable, only results significant for OS are displayed.

		Overall Survival				Progression-Free Survival			
Variables		HR	95% CI	*p*-Value	Adjusted *p*-Value	HR	95% CI	*p*-Value	Adjusted *p*-Value
Clinical	WHO performance status	2.59	[1.4; 4.86]	0.003	0.02	2.28	[1.16; 4.5]	0.02	0.06
	Stage	3.3	[1.58; 6.88]	0.001	0.01	3.77	[1.75; 8.13]	<1.10^−3^	0.007
	Lung Metastasis	0.38	[0.18; 0.79]	0.01	0.02	0.87	[0.44; 1.73]	0.69	0.69
	Liver Metastasis	2.51	[1.38; 4.55]	0.002	0.01	1.82	[0.97; 3.41]	0.06	0.12
Genomic	Ploidy > 2.4	3.2	[1.59; 6.47]	0.001	0.007	1.77	[0.84; 3.72]	0.14	0,44
	Clonality > 1	0.34	[0.17; 0.65]	0.001	0.007	0.48	[0.24; 0.95]	0.03	0,38
	CNV signature 1 >86.6%	0.47	[0.24; 0.93]	0.03	0.09	0.79	[0.39; 1.63]	0.53	0.77
	CNV signature 5 >4.7%	2.62	[1.46; 4.7]	0.001	0.007	0.96	[0.52; 1.78]	0.9	0.9
Pathways	Calcium	0.39	[0.14; 1.09]	0.07	0.42	0.48	[0.17; 1.31]	0.15	0.54
	Class I MHC	2.86	[0.86; 9.51]	0.09	0.42	1.11	[0.15; 8.24]	0.92	0.93
	MAPK	4.87	[1.62; 14.6]	0.005	0.07	4.58	[1.5; 13.9]	0.007	0.15
	NHEJ	3.25	[0.99; 10.67]	0.05	0.41	0.45	[0.06; 3.31]	0.43	0.76
	p53	1.61	[0.9; 2.87]	0.1	0.45	0.9	[0.5; 1.58]	0.62	0.87
	Spliceosome	6.28	[2.31; 17.13]	0.0003	0.009	3.19	[1.1; 9.3]	0.03	0.24
	VEGF	2.41	[0.93; 6.22]	0.07	0.41	3.7	[1.36; 10.06]	0.01	0.15

*p*-values of log-rank test were adjusted using Benjamini–Hochberg FDR (false discovery rate) correction; *p*-values less than or equal to 0.1 were considered significant. HR: hazard ratio; CI: confidence interval; TMB: tumor mutational burden; HRD: homologous recombination deficiency; CNV: copy number variant; MHC: major histocompatibility complex; MAPK: mitogen-activated protein kinase; NHEJ: non-homologous end-joining; VEGF: vascular endothelial growth factor.

## Data Availability

Data are available from authors upon reasonable request.

## Supplementary Materials

The following are available online at https://www.mdpi.com/article/10.3390/cancers13081851/s1. Figure S1: Flowchart of the different models estimated in this study; Figure S2: Mutational signature analysis of whole exome sequencing data from the whole population of pancreatic tumors; Figure S3: Analysis of homologous repair aberrations, SNV or CNV signatures 3 and HRD score; Figure S4: Validation of the overall survival models in the gemcitabine group; Table S1: Description of treatment based on molecular tumor board recommendation; Table S2: Hazard ratio with 95% confidence intervals for variables selected through bootstrap strategy for each of the models, estimated using multivariate Cox models with lasso penalty; Table S3: Description of genes involved in signaling pathways of interest.
