# Supplementary material for: Using Exome Sequencing to Improve Prediction of FOLFIRINOX First Efficacy for Pancreatic Adenocarcinoma
Source: Cancers (Basel). 2021 Apr 13;13(8):1851. doi: 10.3390/cancers13081851 (PMC8070262; doi:10.3390/cancers13081851)
Supplement: Supplementary file 1 [file cancers-13-01851-s001.zip › Supplementary files/Supp_Figures2.pdf]

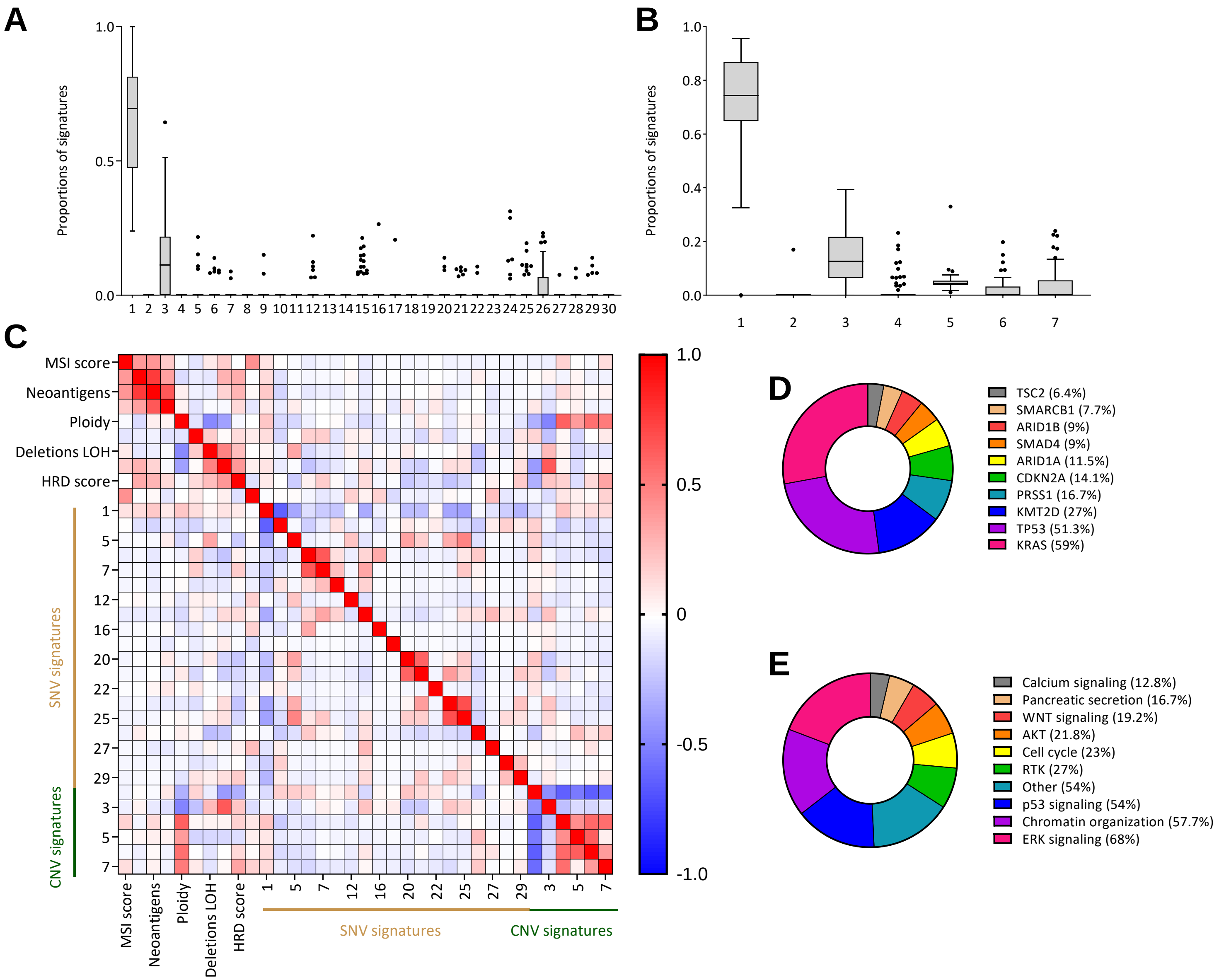

Supplementary Figure 2: Mutational signature analysis of whole exome sequencing data from the whole population of pancreatic tumors.

A) Boxplots showing the proportion of each SNV signature for all patients. B) Boxplots showing the proportion of each CNV signature for all patients. C) Heatmap showing the correlation matrix between genomic variables; correlations were calculated with Pearson's correlation coefficient. D) Distribution of the 10 most frequent genes in the whole cohort (percentage of patients with this gene). E) Distribution of the 10 most highly represented signaling pathways in the whole cohort (percentage of patients with this signaling pathways).
