# Supplementary material for: Using Exome Sequencing to Improve Prediction of FOLFIRINOX First Efficacy for Pancreatic Adenocarcinoma
Source: Cancers (Basel). 2021 Apr 13;13(8):1851. doi: 10.3390/cancers13081851 (PMC8070262; doi:10.3390/cancers13081851)
Supplement: Supplementary file 1 [file cancers-13-01851-s001.zip › Supplementary files/Supp_Figures3.pdf]

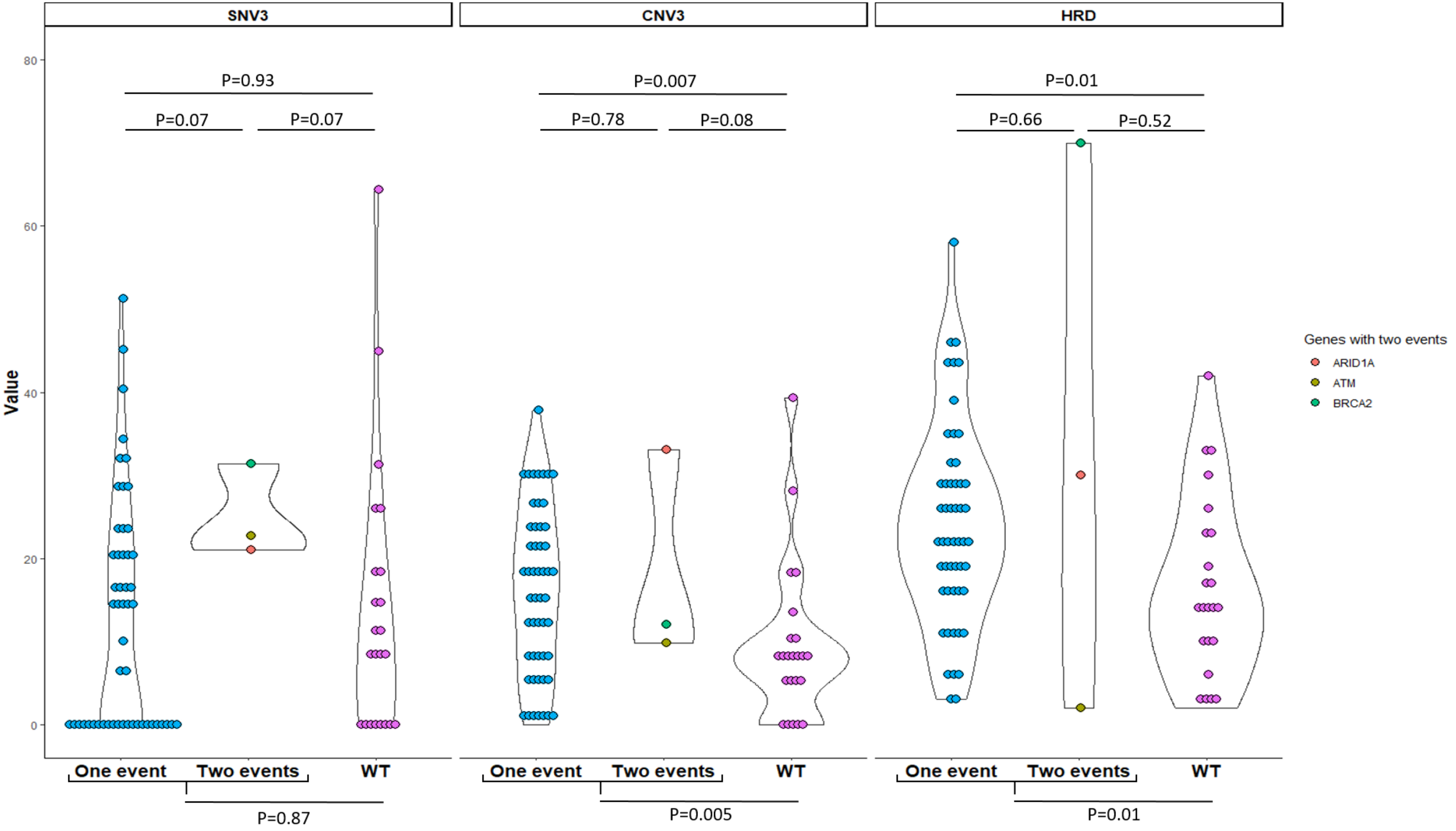

Supplementary Figure 3: Analysis of homologous repair aberrations, SNV or CNV signatures 3 and HRD score.
Violin plots showing the proportion (%) of the SNV and CNV signatures 3 and HRD score for HRD-altered (with one or two events) and wild-type (WT) patients. An event is defined as somatic or germline mutation, or LOH on ARID1A, ATM or BRCA2 genes. "One event" category includes patients with one event on at least one gene. "Two events" category includes patients with two events on at least one gene. Top p-values correspond to Wilcoxon test between each of the three categories; bottom, p-values correspond to Wilcoxon tests between HRD-altered and wild-type (WT) patients.
