# Supplementary material for: Using Exome Sequencing to Improve Prediction of FOLFIRINOX First Efficacy for Pancreatic Adenocarcinoma
Source: Cancers (Basel). 2021 Apr 13;13(8):1851. doi: 10.3390/cancers13081851 (PMC8070262; doi:10.3390/cancers13081851)
Supplement: Supplementary file 1 [file cancers-13-01851-s001.zip › Supplementary files/Supp_Figures4.pdf]

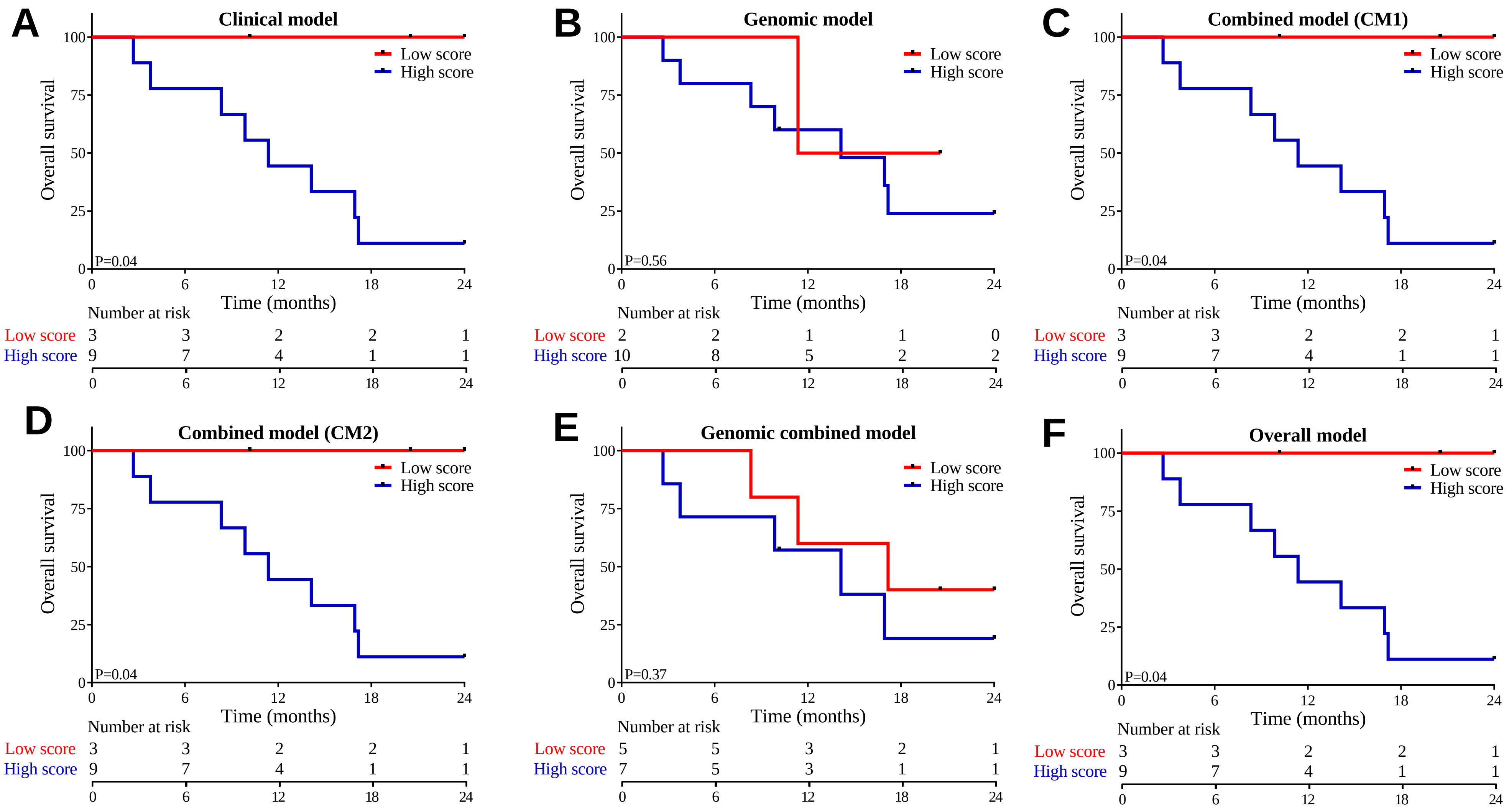

Supplementary Figure 4: Validation of the overall survival models in the Gemcitabine group.
Kaplan-Meier curves for overall survival, with patients stratified according to A) clinical score, B) genomic score, C) combined 1 score (clinical and genomic variables), D) combined 2 score (clinical variables and pathways), E) genomic part of the global model (genomic variables and pathways) and F) overall score (clinical, genomic variables and pathways). Note that curves were not represented for the pathway model because of lack of mutation in the selected pathways.
