# Supplementary material for: Using Exome Sequencing to Improve Prediction of FOLFIRINOX First Efficacy for Pancreatic Adenocarcinoma
Source: Cancers (Basel). 2021 Apr 13;13(8):1851. doi: 10.3390/cancers13081851 (PMC8070262; doi:10.3390/cancers13081851)
Supplement: Supplementary file 1 [file cancers-13-01851-s001.zip › Supplementary files/Supplementary Table 1.pdf]

Supplementary Table 1: Description of treatment based on Molecular Tumor Board recommendation

SAMPLE	MUTATION	NUCLEOTIDIC VARIANT	PROTEIC VARIANT	IMPACT	MUTATION SOMATIC : 1 GERMLINE : 2	BIALLELIC LOSS YES : 1 NO : 2	TREATMENT (line)	PFS (months)
**1**	BRCA2	c.4284dupT	p.Gln1429SerfsTer9	Loss of function	2	2	Olaparib (3)	0.37
**2**	BRCA2	c.273C>A	p.Tyr91Ter	Loss of function	2	1	Clinical trial olabarib durvalumab tremelimumab	7.6
**3**	BRCA1	c.5295A>C / c.4956G>A	p.Glu1765Asp / p.Met1652lle	unknown	2	2	Olaparib (2)	0.53
**4**	BRIP1	c.3G>A	p.Met1 ?	unknown	2	2	Olaparib (4)	0.9
**5**	CDKN2A	c.47_50delTGGC	p.Leu16ProfsTer9	Loss of function	1	2	palbociclib trametinib (2)	1.4
**6**	TP53	c.498dupA	p.Gln167ThrfsX14	Loss of function	1	1	Sorafenib (4)	0.57
**7**	KRAS	c.182A>G	p.(Gln61Arg)	activating	1	2	panitumumab trametinib (4)	0.7
**8**	BRCA2	c.9976A>T	p.Lys3326X	Loss of function	2	2	Olaparib (2)	2.03
**9**	ARID1A	c.2291delC	p.Gln766SerfsTer67	Loss of function	1	2	Everolimus (3)	0.23
**10**	STK11	c.662C>T	p.Pro221Leu	Unknown	2	1	Everolimus (3)	0.63
**11**	KRAS	C.35G>T	p.(Gly12Val)	Activating	1	2	panitumumab trametinib (3)	2.03
