# Supplementary material for: Using Exome Sequencing to Improve Prediction of FOLFIRINOX First Efficacy for Pancreatic Adenocarcinoma
Source: Cancers (Basel). 2021 Apr 13;13(8):1851. doi: 10.3390/cancers13081851 (PMC8070262; doi:10.3390/cancers13081851)
Supplement: Supplementary file 1 [file cancers-13-01851-s001.zip › Supplementary files/Supplementary Table 2 .pdf]

Supplementary Table 2 : Hazard ratio with 95% confidence intervals for variables selected through bootstrap strategy for each of the models, estimated using multivariate Cox models with lasso penalty.

VARIABLES		HR	95% CI
**CLINICAL MODEL**	WHO Performance status	2.85	[2.68 ; 3.06]
	Stage	2.36	[2.27 ; 2.46]
	Liver Metastasis	1.78	[1.71 ; 1.86]
	Lung Metastasis	0.31	[0.29 ; 0.33]
**GENOMIC MODEL**	Ploidy > 2.4	2.27	[2.15 ; 2.37]
	Clonality > 1	0.48	[0.46 ; 0.5]
	CNV signature 1 > 86.6%	0.64	[0.61 ; 0.67]
	CNV signature 5 > 4.7%	1.34	[1.32 ; 1.35]
**COMBINED MODEL 1**	WHO Performance status	2	[1.92 ; 2.12]
	Stage	2.1	[2.02 ; 2.21]
	Liver Metastasis	1.48	[1.44 ; 1.54]
	Lung Metastasis	0.49	[0.46 ; 0.51]
	Ploidy > 2.4	1.35	[1.33 ; 1.37]
	Clonality > 1	0.62	[0.59 ; 0.63]
	CNV signature 1 > 86.6%	0.89	[0.86 ; 0.91]
	CNV signature 5 > 4.7%	1.33	[1.32 ; 1.35]
**PATHWAYS MODEL**	Calcium	0.65	[0.51 ; 0.72]
	NHEJ	1.38	[1.04 ; 2.07]
	Spliceosome	3.73	[2.96 ; 5.4]
**COMBINED MODEL 2**	WHO Performance status	2.2	[1.9 ; 2.55]
	Stage	1.78	[1.63 ; 1.95]
	Liver Metastasis	1.52	[1.37 ; 1.57]
	Lung Metastasis	0.43	[0.37 ; 0.5]
	Calcium	0.58	[0.47 ; 0.67]
	NHEJ	1.67	[1.13 ; 2.26]
	Spliceosome	2.91	[2.26 ; 3.57]
**OVERALL MODEL**	WHO Performance status	1.93	[1.74 ; 2.18]
	Stage	1.81	[1.65 ; 2.05]
	Liver Metastasis	1.38	[1.31 ; 1.47]
	Lung Metastasis	0.51	[0.44 ; 0.57]
	Ploidy > 2.4	1.24	[1.18 ; 1.33]
	Clonality > 1	0.71	[0.69 ; 0.73]
	CNV signature 1 > 86.6%	0.92	[0.86 ; 0.96]
	CNV signature 5 > 4.7%	1.29	[1.27 ; 1.3]
	Calcium	0.59	[0.48 ; 0.69]
	NHEJ	1.26	[1 ; 1.69]
	Spliceosome	2.26	[1.84 ; 2.82]

HR: hazard ratio; CI: confidence interval; CNV: copy number variant; NHEJ: non-homologous end-joining.
