# Supplementary material for: Using Exome Sequencing to Improve Prediction of FOLFIRINOX First Efficacy for Pancreatic Adenocarcinoma
Source: Cancers (Basel). 2021 Apr 13;13(8):1851. doi: 10.3390/cancers13081851 (PMC8070262; doi:10.3390/cancers13081851)
Supplement: Supplementary file 1 [file cancers-13-01851-s001.zip › Supplementary files/Supplementary Table 3 .pdf]

Supplementary Table 3: Description of genes in interest signaling pathways

Signaling Pathways	*Genes*
Calcium signaling	CYSLTR2, **GNAS, GRIN2A**, PRKACA, PRKACB, **GNA11**, **GNAQ**, ITPKA, ITPKB, ITPKC, ITPR1, ITPR2, ITPR3, PLCB1, PLCB2, PLCB3, PLCB4, PLCG1, **PLCG2**, PRKCA, PRKCB, PRKCG, PTK2B
Pancreatic secretion	**PRSS1**
WNT signaling	LRP1B, RNF43, **APC**, APC2, **AXIN1**, AXIN2, CTNNB1, CTNNBIP1, CTNND1, DAAM1, DAAM2, DKK1, DKK2, DKK3, DKK4, DKKL1, DVL1, DVL1P1, DVL2, DVL3, FBXW11, FBXW2, FBXW4, FRZB, FST, FZD1, FZD10, FZD2, FZD3, FZD4, FZD5, FZD6, FZD7, FZD8, FZD9, GSK3A, GSK3B, GSKIP, **LRP1**, ,RP4, LRP5, LRP5L, LRP, NKD1, NKD2, PORCN, PRICKLE1, RAC1, RAC2, RAC3, RHOA, RHOU, RHOV, RUVBL1, RYK, SENP2, SFRP1, SFRP2, SFRP4, SFRP5, SKP1, TCF15, TCF23, **TCF3**, TCF4, TCF7, TCF7L1, **TCF7L2**, TDGF1, TGFB1I1, VANGL2, WIF1, WISP1, WISP2, WISP3, WNT1, WNT10A, WNT10B, WNT11, WNT16, WNT2, WNT2B, WNT3, WNT3A, WNT4, WNT5A, WNT5B WNT6, WNT7A, WNT7B, WNT8A, WNT8B, WNT9A, WNT9B, CREBBP, EP300, SOX17
AKT	AKT1, AKT1S1, **AKT2**, AKT3, GSK3b, **MTOR**, PDPK1, **PIK3CA**, PIK3CB, **PIK3CD**, PIK3CG, **PIK3R1**, PIK3R2, **PIK3R3**, PIK3R4, PIK3R5, PIK3R6, **PTEN**, TSC1, **TSC2**
Cell cycle	**ABL1**, ANAPC2, AURKA, MDM2, MYC, **PPP2R1A**, SRC, **XPO1**, BUB1, BUB1B, BUB3, CCNA1, CCNA2, CCNB1, CCNB2, CCNB3, CCNC, CCND1, **CCND2,** CCND3, CCNE1, CCNE2, CCNF, CCNG1, CCNG2, CCNH, CCNI, CCNI2, CCNJ, CCNJL, CCNK, CCNL1, CCNL2, CCNO, CCNT1, CCNT2, CCNY, CCNYL1, CCNYL3, CDC14A, CDC20, CDC25A, CDC25B, CDC45, CDC6, CDC7, CDH1, CDK1, CDK2, CDK4, CDK6, CDK7, **CDKN1A**, CDKN1B, CDKN1C, **CDKN2A**, CDKN2B, **CDKN2C**, CDKN2D, CIB2, CRIP2, E2F1, E2F2, E2F3, E2F4, E2F5, ELL, ESPL1, FZR1, HDAC9, KITLG, MAD1L1, MAD2L1, MXD1, MXI1, MYT1, NOLC1, PKMYT1, PLK1, PMEPA1, PTTG1, RAD21, **RB1**, RBL1, RBL2, SKP2, SLC12A9, SMC2, STAG1, STAG2, TFDP1, TFDP2, TTK, YWHAQ

RTK	AATK, **ALK**, AXL**, CSF1R**, DDR1, **DDR2**, EGFR, EPHA1, EPHA10, EPHA2, EPHA3, EPHA4, **EPHA5**, EPHA6, **EPHA7**, EPHA8, **EPHB1**, EPHB2, EPHB3, EPHB4, EPHB6, **ERBB2**, **ERBB3**, ERBB4, **FGFR1**, FGFR2, FGFR3, **FGFR4**, **FLT1**, FLT3**, FLT4**, **IGF1R**, **INSR**, INSRR, **KDR**, KIT, LMTK2, LTK, MET, **MPL**, MERTK, MST1R, MUSK, **NTRK1,** NTRK2, **NTRK3**, **PDGFRA**, **PDGFRB**, PDGFRa, PDGFRb, PTK7, **RET**, ROR2, RORA, ROS1, RYK, SLTM, STYK1, **TEK**, TIE1, TYRO3
P53 signaling	**ATM**, **ATR**, CHEK1, CHEK2, FOXA1, MDM4, PPM1D, SESN1, SESN2, SESN3, **TP53**
Chromatin signaling	**ARID1A**, **ARID1B**, **ARID2**, **ASXL1**, **ASXL2**, BRD4, **DNMT1**, DNMT3A, DNMT3B, **DOT1L**, **ERG**, **EZH1**, EZH2, **KMT2A**, KMT2B, **KMT2C**, **KMT2D**, **NCOR1**, **NPM1**, NSD1, **PBRM1**, **SETD2**, **SMARCA4**, TET1, **TET2**, **SMARCB1**
ERK signaling	**BRAF**, DUSP1, DUSP10, DUSP16, DUSP2, **DUSP4**, DUSP5, DUSP6, DUSP7, DUSP8, DUSP9, HRAS, IGF1, **KRAS**, **MAP2K1**, MAP2K2, MAPK1, MAPK3, MOS, **NF1**, NRAS, PEA15, **PTPRD**, RAF1, RAP1A, RAP1B, RAPGEF2, RASA1, RASA2, RASGRP1, RASGRP2, RASGRP3, RASGRP4, RPS6KA1, RPS6KA2, RPS6KA3, SOS1, SOS2, SPRY1, SPRY2, SPRY3, SPRY4

Genes present in our cohort are in bold.
