# Supplementary figures and images for: Using Exome Sequencing to Improve Prediction of FOLFIRINOX First Efficacy for Pancreatic Adenocarcinoma
Source: Cancers (Basel). 2021 Apr 13;13(8):1851. doi: 10.3390/cancers13081851 (PMC8070262; doi:10.3390/cancers13081851)

Supplementary Figure 1: Flowchart of the different models estimated in this study.

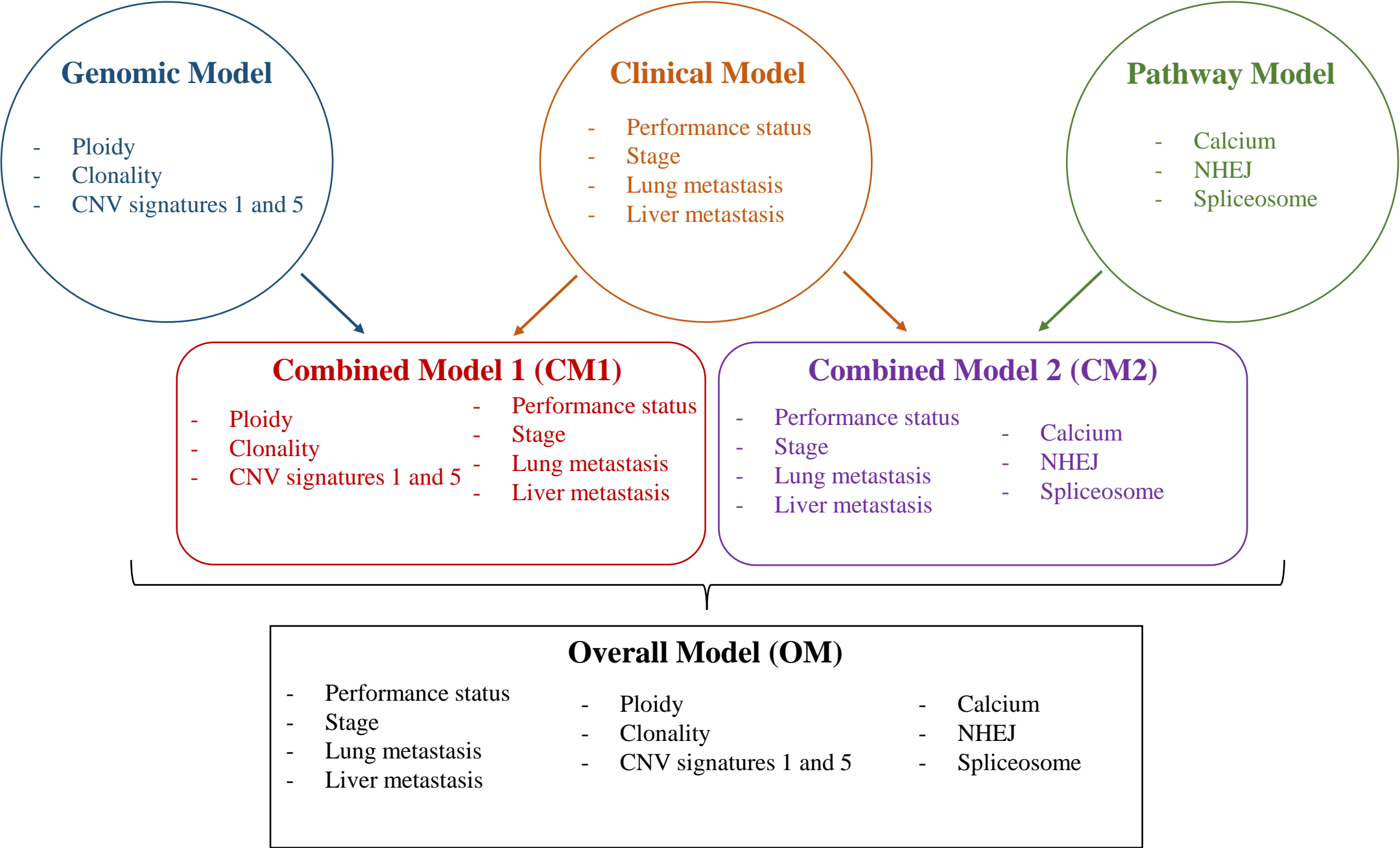

Supplement: Supplementary file 1 [file cancers-13-01851-s001.zip › Supplementary files/Supp_Figures1.pdf]
